# India’s foreign economic policy under Modi: negotiations and narratives in the WTO and beyond

**DOI:** 10.1057/s41311-020-00275-z

**Published:** 2021-02-02

**Authors:** Amrita Narlikar

**Affiliations:** German Institute for Global and Area Studies (GIGA), Hamburg, Germany

**Keywords:** India, Foreign economic policy, World Trade Organization, Negotiations, Narratives, Weaponized interdependence

## Abstract

For all the complexities of India’s politics, Prime Minister Narendra Modi seemed to have his economic path cut out for him. His ‘Achche din aane waale hein’ (good days are coming) campaign, which had won him a resounding election victory in 2014 for his first term, suggested that Modi’s primary goal was growth and development for his country and people, and hence also an agenda of economic reform. Focusing specifically on India’s negotiations in the context of the WTO, I show in this paper that India has continued to hold on to its former trade policy priorities and negotiation positions and adopted even more hard-line positions in some cases. Interestingly though, the same policy priorities and negotiation patterns that had ill-served India in the past may now no longer be a liability. This is only in part a credit to the Modi administration per se. Rather, it is mainly due to the rise of the phenomenon of “weaponized interdependence”, which in turn legitimizes—sometimes even necessitates—the securitization of foreign economic policy, and more specifically, trade politics. Taken in this changing context and as other countries also adopt a more market-cautionary approach, India’s historic and oft-reviled trade scepticism and reluctance to integrate in global value chains may yet allow it to have the last rhetorical laugh.

## Introduction

For all the complexities of Indian politics, Prime Minister Narendra Modi’s path on economic policy (domestic and foreign) seemed to be clearly cut out. His ‘Achche din aane waale hein’ (good days are coming) campaign, which had won him a resounding election victory in 2014 for his first term, suggested that Modi’s primary goal was growth and development for his country and people, and hence also an agenda of economic reform. In response to the election results, the stock market had surged excitedly (Kumar 2014; Riley 2014) under the expectation that the new administration would pursue an aggressive agenda for economic reform domestically and match this by a foreign economic policy that would be enthusiastically pro-trade. This, in turn, would translate into India taking on a more proactive role in the World Trade Organization and perhaps also signing new regional and bilateral trade agreements. A closer analysis, however, reveals quite the opposite.

Focusing specifically on India’s negotiations in the context of the WTO, I show in this paper that India has continued to hold on to its prior policies and negotiation positions under Modi’s premiership and adopted even more hard-line positions in some cases. Interestingly though, the same policy priorities and negotiation behaviour patterns that had ill-served India in the past may no longer be a liability. This is only in part a credit to the Modi administration per se. Rather, it is mainly due to the rise of the phenomenon of weaponized interdependence (Farrell and Newman 2019). As recent years have begun to demonstrate and contra the assumptions that had underpinned the post-war multilateral order, globalization is not always a benign force for prosperity and peace; the very ties that were supposed to bind nations into a liberal peace can sometimes be exploited for security purposes, i.e. ‘weaponized’. The fact that global trade is now conducted via closely integrated value chains allows unprecedented power to a few countries that control network hubs of production and increases the vulnerabilities of other players. The security threats posed by this deep economic integration legitimize—sometimes even necessitate—the securitization of foreign economic policy, and more specifically, trade politics. Taken in this changing context, India’s historic and oft-reviled market scepticism looks far from being an outlier; indeed, it may even appear to be a foresighted response to new challenges.

My paper proceeds in five sections. In the first section, I briefly outline the main economic achievements of the Modi regime in the last six years and also point to the limitations. While the successes are not to be scoffed at, they are usually within the realm of domestic economic policy; when it comes to foreign economic policy, the record is less impressive (plus there is much that still remains to be done also on the domestic economic front). In the second section, I suggest that an overlooked but important explanation for the differential performance lies in the field of narratives. I offer a brief theoretical overview of the concept and posit that the most significant changes occurred in areas where the government constructed new and winning narratives. In the third section, I focus on Modi’s foreign economic policy within the context of the World Trade Organization (WTO) and compare it to previous eras. The reason for my choosing to concentrate on this particular aspect of India’s foreign economic policy derives from India’s long-standing commitment to multilateralism over the decades and also India’s growing voice within the WTO. Additionally, trade liberalization is seen as one of the less controversial areas of economic reform (in contrast to opening of financial markets or aid policies) for governments that wish to prioritize growth and development. The multilateral trade regime plus the nature of the issue together offer a context where we would expect to see the most obvious and immediate signs of trade liberalization and proactive agenda-setting. As such, it is an ‘easy’ test-case; if India’s negotiation priorities and positions remain unchanged even in this relatively amenable setting and in a less controversial policy field, we would have grounds to argue that not much is likely to change in India’s foreign economic policy in other less conducive (e.g. bilateral) settings. I show that India’s trade policy priorities, negotiation positions, and narratives today show some important continuities, not only in relation to the years immediately preceding the Modi years, but also with the previous decades. In the fourth section, I offer an explanation for the persistence of India’s old trade narrative and indeed its hardening in some instances. I argue that a major factor lies in the international and regional context—typified by the phenomenon of ‘weaponized interdependence’—that legitimizes old narratives of protectionism and strengthens them further. In the fifth section, I offer some conclusions.

## Major economic trends under Modi’s leadership

When Modi came to power in 2014 as India’s Prime Minister, expectations were high. Chatterjee-Miller (2014), for example, writing just before the elections, offered two interesting quotes to illustrate this. She wrote:

In the event of a BJP government, the assessment of both foreign and Indian officials is that Modi is a very astute leader. In conversations, they seem very hopeful at the prospect of him becoming prime minister. ‘He is very dynamic,’ the senior diplomat to India said of Modi. ‘What worked in Gujarat will translate to the national scene. He will take risks.’ The former foreign secretary concurs. ‘He understands globalization, will not be blindsided, and he will be influenced by the overseas Gujarati diaspora, who are very active and networked.’ And, this official added, Modi ‘respects the bureaucracy,’ which bodes well for a politician with little foreign policy experience.

Note that both quotes hint at high expectations particularly on the economic front. There were good reasons for this optimism; besides his historic association with Gujarat’s economic success, the Prime Minister was elected on a manifesto of ‘economic revival’. A programme of ‘economic revival’ was high up in its ambitions, which involved job creation, combating corruption, making India a manufacturing hub, agricultural growth, tax reform, addressing inequality and extreme poverty, ensuring food security, make ‘doing business’ in India easy, infrastructure development, and more (BJP 2014). Together, all the aspirations and promises made for a tall order. And while no government could realistically fulfil all the goals that the manifesto outlined, let alone a chaotic and colourful democracy like India, Modi 1.0 deserves credit for many achievements within the rubric of his ambitious goals.

The Modi government, in its first term, had taken on some challenging tasks, including the introduction of a Goods and Services Tax (seen as too hot a potato by previous governments). The GST seeks to unify and regularize India’s fragmented and complex tax system (World Bank 2018). Infrastructure received a boost; for instance, the inclusion of some 90% of India’s population into the electric grid was no mean feat. Even though the election campaign in 2019 was fought on a more nationalistic platform, which emphasized the ability of the BJP to protect the country from threats in the neighbourhood, it did not shy away from laying claim to its significant successes during Modi’s first term. The government had set up 355 million Indians with bank accounts for the first time. Its Swacch Bharat Abhiyan had built close to 100 million toilets. The Prime Minister’s Jan Arogya Yojana aimed for universal healthcare and had reached 1.8 million beneficiaries by the end of Modi’s first term. From the provision of Liquified Petroleum Gas (LPG) to 72 million households, to providing 100 million individuals with small loans to promote an entrepreneurial culture, there were several ‘touchpoints’ that formed a part of Modi’s delivery of economic governance, which appealed to the electorate (Chikermane 2019a). Its services sector leapfrogged ahead, offering smooth and swift visa services, passport services for its own citizens, ease of digital payments for retail purposes and more (Halan 2019). The promise of ‘less government, more governance’ also seemed to pay off in terms of India’s improving attractiveness for investors: after years of having occupied embarrassingly low ranks in the World Bank’s Ease of Doing Business index, India jumped up a remarkable 65 places under Modi. The markets rewarded Modi’s return to power with elation (Economic Times 2019).

Inevitably, there were also some misses. Some were perhaps just errors of judgement. The demonetization drive was a case in point. Different motivations have been attributed to this effort, including an attempt to snuff out corruption, signal (internally and externally) a strong commitment to reform, stamping out counterfeit currency that was believed to be funding terrorism, and more (Subramanian 2018). The move ended up producing extreme disruption and is reported to have cost India a significant deceleration in growth and some 1.5 million job losses (Safi 2018; also Rajan 2019). In other areas, systemic failures were rampant and persistent.

Even before the COVID19 pandemic hit, there was recognition that India would need to ‘take its growth trajectory to the next level’ (Mohan 2019), if it really wanted to eliminate poverty and move into the category of upper middle-income categories. In fact, reporting just a few months after Modi’s electoral landslide victory, the Economist (2019a) summarized the state of affairs in the following words:

Two months later, the elation is gone. Despite an uptick in August, Mumbai’s Sensex stock index is about as close to October’s lows as it is to June’s highs. In July foreigners pulled more money out of Indian equities than they put in. India’s cautious business press has begun to criticise the government. So too, even more gingerly, have its cowed business leaders. ‘There is no demand and no private investment,’ groused Rahul Bajaj, chairman of Bajaj Auto, a motorcycle-maker, at its annual meeting in late July. ‘So where will growth come from?’ The remark, widely interpreted as a swipe at Mr Modi, encapsulates Indian business’s disenchantment with the man they once regarded as their champion.

Many of the problems are deep-rooted and structural. Agriculture has remained a particularly difficult area; annual growth rate in this sector fell to less than 3% between 2014 and 2019. There is recognition that if India is to attain the high growth rates it aspires to, productivity in a sector that occupies over 50% of its population will have to increase. ‘Serial market failures’ (Chikermane 2019b) have afflicted this sector, including inadequate food supply chains, transportation, and storage facilities and more (Mohan 2019). Manufacturing is recognized as a critical to growth, but remains beset with problems ranging from antiquated labour laws, land acquisition laws, what Chikermane and Agrawal (2020) describe as ‘a regulatory cholesterol that has been thickening and slowing the free flow of business, simultaneously weakening and feeding off the economic enthusiasm of entrepreneurs’. They illustrate what this adds up to with the following example:

…a small company with a single plant and up to 500 employees faces 23 licences or registrations, more than 60 laws, more than 750 compliances and needs to make more than 120 filings a year or one every three days. The numbers for a medium sized company with six plants and up to 5,000 employees are respectively 98, 135, 5,500 and 530 (or about three filings every two days). And for a large company of 11 plants and up to 10,000 employees, the numbers are 163, 210, 9,500 and 940 (or about five filings every two days).

Monika Halan (2020) notes a similar pattern: ‘Our laws, bureaucracy and rules come and stand in the way—they are inflexible and coercive’. Nor have these challenges been limited to India’s agenda for domestic economic reform.

India’s slowness to reform and modernize its agriculture sector, or indeed produce the necessary leaps in its manufacturing sector, have been accompanied by considerable reserve in opening up its own market or accepting new commitments in the area of international trade—both at the regional and multilateral levels. India’s characteristic suspicion of regional trade agreements (recall, for instance, the EU-India FTA negotiations that were started in 2007, and then suspended in 2013, i.e. prior to Modi’s arrival on the scene persisted in the Modi years, see European Commission 2020) persisted and possibly deepened. India’s negotiations with New Zealand for an FTA had been launched in 2010; the last formal round of negotiations took place in 2015, with no conclusion. The Canada-India FTA negotiations, also launched in 2010, have similarly been put on the back burner. A study from NITI Aayog (a think-tank of the Indian government) offered analytic grounds for maintaining scepticism of existing FTAs (Saraswat et al. 2019). This scepticism played out dramatically in the negotiations over the Regional Comprehensive Economic Partnership; 15 of the 16 parties signed on to the deal in November 2019, but India chose to stay out (Economist 2019b). In doing so, it attracted approval from senior commentators within the country (e.g. Dhar 2019). India’s existing dependence on China for imports (and lack of reciprocal market access for its exports) was cited as an important strategic consideration for rejecting RCEP; India’s Minister for Commerce and Industry lauded Modi for ‘his bold and courageous decision to not join RCEP, since it was against our economic interests and national priorities’ (Goyal 2019). The Economist (2019a) summarized India’s position on multilateral trade with a short and damning verdict on the Modi government’s record in its first term: ‘Rather than promoting trade, it scrapped existing bilateral deals, raised tariffs and sparred with the WTO’. It was becoming increasingly clear that Modi’s business-friendly approach in Gujarat would not automatically result in a market-friendliness at the national level (Chatterjee 2020).

## Narratives and foreign economic policy

Difficulties to carry out deep structural economic reform are perhaps to be expected. The federal system exacerbates the complexities of implementing the Goods and Services Tax; modernization of land acquisition laws and rationalization of small-holdings comes loaded with histories of colonial and post-colonial deprivation; change in labour laws has been the *bête noire* for many previous governments as well; improvements in infrastructure to facilitate a manufacturing boost and a second agricultural revolution do not happen overnight or even over a single term of an ambitious government. But foreign economic policy—and especially the politics of international trade—should be different.

We would expect a growth-oriented government to use an organization like the WTO to readily embrace binding tariff commitments, and thereby not only offer predictability to trading partners but also lock in the reform process at home. Further, by working through an institution like the WTO, countries can secure the gains of unilateral trade liberalization (that mainstream economists advocate) as well as access to the markets of others. This allows the mobilization of export-oriented interest-groups and can help balance them out against import-competing protectionist interest-groups. A government pushing for reform can even use the cognate international organization to shift the blame for tough measures (such as the WTO and the mandatory protection of Intellectual Property Rights that it requires from its members). In fact, however, this is not how the reform process has played out in India. Even while successfully bringing about some important policy changes, which have a positive impact on the economic lives of its people, the Modi government has not played the two-level game to its advantage. And while there are several explanations as to why the government has achieved success in some areas and not in others (e.g. Mohan 2019), an important—and usually overlooked—explanation is that of narratives.

To scholars in the Humanities, it has been obvious for a long time that stories matter (e.g. Macintyre 1981; Fisher 1984). Complementing this scholarship has been research in the social sciences, which has long highlighted the need to go beyond conventional notions of rationality to understand drivers for decision-making among individuals and groups. In 1936, John Maynard Kaynes had argued that under conditions of uncertainty, decisions are not ‘the outcome of a weighted average of quantitative benefits multiplied by quantitative probabilities’; rather, decisions ‘can only be taken as a result of animal spirits’. Simon (1955, 1972) had developed the notion of ‘bounded rationality’; Kahneman and Tversky (1979) had challenged traditional expected utility theory and instead offered ‘prospect theory’ as an alternative model in the field of behavioural economics. Parallel developments had taken place also in political science: Allison (1971) had written on the limitations of rational-actor models in decision-making; Janis (1972) on groupthink and Goldstein and Keohane (1993) on identities and ideas in foreign policy and Raymond Cohen on the importance of culture in international negotiations (1991). In the field of International Relations, several schools of thought flourished—ranging from constructivism to sociological institutionalism—focused on the role of norms as a key explanatory variable for international behaviour (e.g. Klotz 1995; Finnemore 1996; Acharya 2004). All these insights were helpful in challenging narrow notions of rationality. But unlike the richly researched higher level of ideas, norms, and identities on the one hand, and the tactical level of framing on the other, the realm of stories had remained largely under-studied in the social sciences. The field of *narrative economics* has shed new light on this subject in recent years.

Robert Shiller (2017), who coined the term ‘narrative economics’, offers the following definition of the field: ‘‘By narrative economics, I mean the study of the spread and dynamics of popular narratives, the stories, particularly those of human interest and emotion, and how these change through time, to *understand* economic fluctuations’, A narrative, in turn, is ‘a simple story or easily expressed *explanation* of events that many people want to bring up in conversation or on news or social media because it can be used to *stimulate the concerns or emotions* of others, and/ or because it appears to advance *self-interest*’ (emphasis added). Collier (2016), also contributing to this debate, offers a distinction between three types of beliefs (which, along with networks, constitute ‘culture’): identities (which ‘influence preferences’), narratives (which ‘influence how causal relationships are (mis)understood’), and norms (which ‘determine self-imposed constraints’).^1^ In terms of their ability to trigger ‘rapid change’ by ‘going viral’ (Shiller 2017), Narlikar (2020) argues that narratives are ‘potentially the more responsive and pliable tool for policy intervention and can further contribute to the shaping of identities and norms’.

Narratives provide accessible explanations and legitimation for governments to carry out specific policies and to convince their electorates to abide by them. In some sectors, Modi clearly marked out his terrain as an agent of transformation and developed a narrative accordingly. A nice example of this was in the area of climate change. In this issue-area, Modi developed a powerful green narrative by appealing to ancient Indian traditions. He thereby helped make mitigation and adaptation measures palatable at home and also ensured that India could show unprecedented and constructive leadership in the negotiations over the Paris Agreement (Narlikar 2017). Similarly, by developing pro-poverty-alleviation narratives, the Modi government was able to bring about reform that visibly and positively affected the lives of citizens. It did this by appealing not only to a diffused national interest, but by also targeting the concerns of multiple groups affected by poverty—e.g. minorities, women, extremely poor, in terms of food security, specific ‘backward’ districts and more—which helped unite a diverse set of political economy interests in support for implementing the agenda, plus produced synergies in the areas of business (such as linkage of Aadhar Card numbers with bank accounts, facilitating business transactions via mobile apps). On various aspects of tax reform, the BJP’s manifesto appealed to different interest groups to help create a consensus against ‘tax terrorism’ (‘which not only creates anxiety amongst the business class and negatively impacts the investment climate, but also dents the image of the country’) and in favour of a rationalization and simplification of the tax regime (BJP Election Manifesto 2014). But as the manifesto had also revealed, the Modi regime was not going to be a poster-child for a Washington Consensus style of market opening.

The same manifesto of the BJP, which had showed so much economic ambition, was also the manifesto that had promised to put ‘India First’.^2^ The narrative was not one of reduced protectionism. While paying attention to several important goals (such as cutting ‘red-tapism’), the manifesto stated clearly: ‘We should no longer remain a market for the global industry. Rather, we should become a Global Manufacturing Hub… A strong manufacturing sector will not only bridge the demand–supply gap leading to price stabilization, but also create millions of jobs and increase incomes for the working class. Above all, it will increase the revenue for government and lead to import substitution to bring down the import bill’. That this was not ‘just’ an election promise but a real commitment has been evident over the last years; it is difficult not to see some clear protectionist strains in Modi’s ‘Make in India’ campaign (e.g. Aiyar 2018). And while India is certainly not the only country to be turning inwards in recent years (a Global Trade Alert Report assessing trade policy across countries over three years of populism (2017–2019) noted, ‘the political rhetoric more critical of a liberal trading system witnessed in recent years has translated into greater protectionism and less trade liberalization *worldwide*’, see Evenett and Fritz 2019), India formed a part of this trend with a large number of discriminatory interventions that were put in place. Amidst the Covid19 pandemic, Modi’s narrative has taken an even more defensive and old-fashioned turn. For instance, the ‘Atmanirbhar Bharat Abhiyan’ refers explicitly to ‘self-reliance’, reminiscent of ideas of ‘self-sufficiency’ that had in fact been propagated by Jawaharlal Nehru (Kamal 2020; Singh and Tembey 2020). New narratives of infusing dynamism in certain aspects of the Indian economy (e.g. via greater ‘economic freedom’) are thus interestingly accompanied by alternative and old-fashioned narratives of self-reliance and protectionism. The latter type of narratives appears most explicitly in India’s negotiation positions in the WTO.

## India in the WTO

Modi’s business-friendly approach in Gujarat, plus the promises made in 2014 towards securing growth and poverty alleviation, had led to expectations that India would show increasing willingness to take on new responsibilities in the WTO. Admittedly, the WTO itself was not in its best shape: the Doha Development Agenda (DDA) had been deadlocked for over a decade. And with President Trump’s arrival on the scene in 2017, the challenges facing the organization, and indeed all trading partners of the USA, became harder still. Protectionism rose. But amidst the sound and fury of Trump’s ‘good and easy to win’ trade wars, retaliation, and counter-retaliation, the positions that the Modi administration adopted in the WTO are a better indication of the country’s medium to longer-term goals and expectations. In the first part of this section, I investigate the narrative that the Modi regime has used in the WTO, and how this has translated into India’s negotiation strategy and coalition formations. I do so by focusing on one key area that India has consistently priortized over the decades—Special and Differential Treatment (SDT). In the second part of this section, I delve back into India’s negotiation behaviour in the pre-Modi years. India’s narrative in the WTO, irrespective of changes in governments, parties and ideologies, shows some distinctive continuities over the past decades.

### The Modi era

Three general observations stand out in India’s negotiation behaviour in the WTO during the Modi era thus far. First, the narrative that India uses is a trade-sceptic and defensive one, which prioritises a reform of the international rules (rather than harnessing them, in their existing form, to the country’s advantage). Often, this narrative is framed in terms of powerlessness and victimhood, which sometimes—especially to external observers and its negotiating counterparts—sits at odds with India’s self-perception as a rising power and indeed the status accorded to it in the WTO. Second, the use of this narrative manifests itself also in India’s negotiation strategy. India uses mainly a ‘distributive strategy’ in its negotiations, i.e. tactics such as refusing to make any concessions, threatening to hold others’ issues hostage, issuing threats and penalties, worsening the other party’s best alternative to negotiated agreement (BATNA). Integrative strategies comprise attempts to widen the issue space and explore common solutions, i.e. ‘strategies designed to expand rather than split the pie’ (Odell 2000). Third, the same narrative also influences the company that India keeps in the WTO. For all the pragmatism that its current foreign minister, Dr S. Jaishankar, has espoused, in the trade context we do not see a flexibility of coalitions and alliances. In keeping with tradition, India often works in coalitions of developing countries—sometimes even as a leading member of such coalitions—and thereby relies on the power of collective action as much as its own individual leadership to advance its demands. I illustrate the convergence of all three observations in one area of bargaining in the WTO—Special and Differential Treatment.

Special and Differential Treatment (SDT) is an area where India, along with China, has locked horns with major players, particularly the USA. The issue of ‘graduation’ from SDT status—which allowed developing countries exemptions from some obligations—had always been a bone of contention between the global north and the global south. Especially as India, China, and other players grew economically, demands that they ‘graduate’ out of these special entitlements increased. And while previous US administrations had also raised this issue, the Trump administration has sharpened critique of SDT in an unprecedented way. In a submission to the WTO, the USA argued, ‘Whether the WTO's status quo approach to development status was sensible at its dawn, it makes no sense today in light of the vast changes in development and increasing heterogeneity among Members…’ and called for a reform of the system (WTO, Communication from the US 2019a). Instead of continuing to allow self-selection, the USA proposed a new set of criteria whereby countries could avail themselves of SDT: OECD members or those acceding to become members, G20 members, those ranked as high-income by the World Bank, and those with more than 0.5% of global merchandize trade would no longer qualify (WTO, Communication from the US 2019b). India, alone and in coalitions, has fought against such attempts to redefine the terms of SDT qualification.

India’s negotiation narrative reflects its commitment to SDT. In arguing its case, India has made powerful use of a poverty narrative, that points to the condition of its own poor, besides making a collective point. For instance, its Ambassador to the WTO argued:

In any case, even a quick assessment of numbers highlights the gaping divide between the levels of development in developing Members as compared to those of developed Members. I request the audience to take a moment to consider some startling figures: India is home to 35.6% of the world’s poor compared to 38% in all LDCs put together and 195.9 million or 24% of the world’s undernourished people. During the period between 2010 and 2017, on an average India’s per capita GDP was 2.9% that of the United States. Approximately 61.5% of India’s population is dependent on agriculture for their livelihood, and yet data from 2016 shows that domestic support per farmer in the United States is 267 times that in India. Furthermore, India has 81 times the number of farmers per hectare as compared to the United States. In view of this stark development divide, it would be grossly unfair and iniquitous if India were required to take the same obligations as developed countries. The evidence is on our side, even though the resources and rhetoric may not be! (India, WTO 2019b).

This type of narrative has attracted it great hostility, not only in the Trump era but previously too, especially since India’s inclusion in the BRICS grouping and its recognition as a rising economic power. Even as others—such as Brazil, South Korea, and Singapore—have indicated their willingness to forego, India has persisted in holding on to its claim for SDT.

Second, India has adopted a strict distributive strategy on the issue of SDT. For instance, it described SDT as a ‘*non-negotiable right* for all developing members’ (India, WTO 2019a, emphasis added). To bargain on the presumption that one’s own demands can be non-negotiable is an extreme version of a distributive strategy. It further stated:

In view of the gaping divide between our levels of development, it would be grossly unfair and iniquitous if developing countries were required to take the same obligations as developed countries. Against this backdrop, attempts by the United States, to cherry-pick and employ selective economic indicators to deny the persistent divide between developing and developed Members, are painfully worrisome. Preserving special and differential treatment for all developing countries and LDCs, which is a core principle of the WTO, as well as addressing the asymmetries in Uruguay Round Agreements should be an overriding priority.

Third, note that in making the above argument, India makes the case for the entire collective of developing countries. This—given the right of countries to self-declare their developing country status—means that it implicitly binds itself with China’s cause, which has also insisted on preserving its own right to exercise its SDT status. In other places, its commitment to work with China on SDT has been explicit. For instance, it was a signatory to a joint proposal by 10 countries, which included China, that made an impassioned plaidoyer for SDT (WTO 2019c). In doing so, India has allowed itself to get explicitly ‘hyphenated’ with China—a country which is, in fact, considerably ahead of India in per capita terms (Mohan 2019), is a geostrategic rival, and is also the main target of President Trump’s wrath (e.g. Trump 2019). Were India to disassociate itself from China and argue the case for SDT, it might receive a friendlier hearing from the USA and others. But at least publicly, India has been clear not to signal any breaks from China.^3^ Instead, it has preferred to attract the ire of the USA and other developed countries, rather than risk the unity of the coalitions of the global south that its own narrative has consistently backed.

The use of a distributive strategy, collective action through coalitions of developing countries, and a trade-defensive narrative emphasizing poverty and powerlessness can also be found in other key negotiation areas that form a part of the post-Doha agenda: agriculture, e-commerce, and fisheries subsidies. We see a continued willingness to stall the negotiation process and a readiness to ‘just say no’ (Cohen 2001).

### The pre-Modi years and decades

There is little new in the narrative that India has employed in its trade bargaining in the Modi years thus far. Within the context of the WTO, the pre-Modi years saw India exhibit the same three phenomena: the use of a defensive trade narrative, its manifestation in a strict distributive negotiation strategy, as well as collective action via coalitions global south.

At the WTO’s Bali Ministerial Conference in December 2013—a few months before Modi’s election victory—India had turned out to be the leading voice of opposition against an emerging consensus. Its Minister for Commerce and Industry, Anand Sharma, had made the following statement.

We have a half-baked agricultural package, statements of pious intent for Least Developed Countries (LDCs) and several unresolved issues in the trade facilitation agenda.... None of these texts require the developed countries to make binding commitments for the benefit of developing countries. In contrast, developing countries would be required to undertake significant commitments in trade facilitation. If this imbalance in the Bali package is not redressed, the world at large would accuse all of us of collectively making hollow promises and keeping the tank empty on development content.

Historical imbalances in trade rules must be corrected to ensure a rule-based, fair, and equitable order.

The Doha Round, with its strong development mandate, unambiguously recognized the centrality of food security, livelihood security, and rural development in trade negotiations. It acknowledged the inherent imbalance and asymmetries in trade rules and promised to correct historical distortions.

Agriculture sustains millions of subsistence farmers. Their interests must be secured. Food security is essential for over 4 billion people. I recall the words of Mahatma Gandhi ‘There are people in the world so hungry that God cannot appear to them except in the form of bread’. Unlike other areas, the ‘survival’ aspect of agriculture far outweighs any of its ‘commercial’ aspects.

A trade agreement must be in harmony with our shared commitments of eliminating hunger and ensuring the right to food. These are an integral part of the MDGs.

For India food security is *non-negotiable*. (WTO 2013, emphasis added).

Agreement was reached with great difficulty in Bali, with India attracting much criticism for its nay-saying attitude. This critique too, was not new; in 2008—one of the last turning points in the Doha negotiations when a deal had seemed possible—India had led the opposition and contributed to a major deadlock. It had done this in the name of its own poor farmers and in the name of farmers from other developing countries (Blustein 2009). In 2003, it had asserted the voice of developing countries by leading new coalitions (like the G20, together with Brazil, and also the G33—both on agriculture) and forming ‘alliances of sympathy’ with other coalitions of developing countries; this conference too had ended in deadlock and with a clear message that developing countries would no longer subsume their development needs simply for the sake of trade for its own sake (Narlikar 2020).

In fact, India’s history of firmly standing up for the cause of development, sometimes almost framed in opposition to trade liberalization and through a poverty narrative that asserted the rights of the powerless, goes back not only to the early years of the WTO, but as far back as the early years of the General Agreement on Tariffs and Trade. In 1954, India’s representative to the GATT, Sir Raghavan Pillai, had argued for special treatment for developing countries, using a narrative of powerlessness:

…among the contracting parties there are countries which are industrially and economically advanced and others with a backward economy and a very low standard of living. If we wish to retain both classes of countries within one common fold, there will have to be greater flexibility in the provisions that are to apply to all of them. *Equality of treatment is equitable only among equals. A weakling cannot carry the same load as a giant*.’

…There is no bigger obstacle to the advancement of international trade than the poverty of underdeveloped countries…

International trade is not an end in itself. It is but a means to greater prosperity… The GATT cannot of course solve the kind of problem which underdeveloped countries with their rising populations, and low standards of living have to face. The effort must inevitably be their own. What the GATT can do, and must do, is to give them the fullest scope and freedom to fulfil their economic programmes, which I maintain will bring prosperity not only to them but to all those who trade with them (GATT 1954, emphasis added).

The Modi regime may have brought about some important domestic economic reforms at home. But at least as far as its narrative in trade negotiations is concerned (and indeed its negotiation strategy and coalitions)—which constitute a key aspect of foreign economic policy—there is more evidence of continuity than change. Even when its power has risen, India has been wary of opening its own markets and has continued to rely on narratives of poverty and trade defensiveness.

## Explaining persistent patterns in India’s trade narrative

Why do we see such a persistent pattern in its narrative in the WTO, irrespective of changes in governments, parties, ideologies, and changes in its own power positions? There are several explanations for such patterns, including bureaucratic politics, institutions, entrenched interests, colonial legacies, and negotiation culture (e.g. Chatterjee-Miller and Sullivan de Estrada 2017; Hopewell 2015; Sinha 2016). Insofar as narratives of poverty and powerlessness—at least around the turn of the millennium—turned out to generate some good outcomes for India (for instance via the launch of the Doha Development Agenda, and the transformation of the so-called ‘Old Quad’ into new groupings that including Brazil, India, and other developing countries), it might also be possible to ascribe some of the continuities to policy inertia (Narlikar 2020). But a key additional variable, which needs to be taken into account, is the phenomenon of weaponized interdependence.

Contra the assumptions that had underpinned the post-war economic system, which associated growing economic integration with both prosperity and peace, Farrell and Newman (2019) have identified the phenomenon of weaponized interdependence. The global production of goods and services via integrated value chains, as per this argument, generates hierarchical economic networks. Farrell and Newman argue that states which hold political authority over network hubs, and have domestic institutions that support certain types of strategies, are able to ‘weaponize’ networks of interdependence to their advantage. By gathering or restricting information or economic flows through ‘panopticon’ and ‘choke point’ effects, states located on network hubs can ‘discover and exploit vulnerabilities, compel policy change, and deter unwanted actions’. And while we had seen these pernicious effects of weaponized interdependence play out intermittently in the recent past (e.g. China’s export controls on rare earth minerals, Gavin 2013), the COVID19 pandemic drove home these risks with an unprecedented urgency.

As the pandemic unleashed great death and destruction across countries, countries became patently aware of the dangers that globalization and economic integration potentially posed. Several, for instance, responded by putting up export restrictions on critical medical supplies. This group included the EU, which decided to put up emergency export restrictions on hospital supplies, thereby endangering lifelines for not only non-EU countries but also supply chain disruption on medical equipment for the EU itself (Bown 2020). Recognizing the life-threatening shortages that countries were facing, China stepped in with its coronavirus diplomacy. It offered to sell masks, personal protective equipment, ventilators, and more to countries in need. So, for example when the EU put up export restriction denying access of key supplies to Serbia, China came to its rescue (Walker 2020). That global health chains could indeed be exploited, and vulnerabilities in production could be used for geoeconomic purposes, became clear amidst the pandemic. And this created some reservations towards gung-ho globalization. India had always expressed such reservations, but suddenly India was not alone.

These reservations became evident across countries. Margrethe Vestager, EU Commissioner for competition, for instance, stated that European countries should buy stakes in companies amidst the risk of Chinese takeovers of firms struggling during the pandemic (Espinoza 2020). Japan took the decision to reserve 220 billion yen ($2 billion) from its stimulus package to help its own companies move production from China back to Japan and another 23.5 billion yen to enable Japanese firms to move to third countries (Reynolds and Urabe 2020). India too put up new restrictions that targeted FDI from neighbouring countries, to curb ‘opportunistic takeovers/acquisitions of Indian companies due to the current COVID-19 pandemic’ (Indian Ministry of Commerce and Industry 2020). India’s refusal to sign RCEP also makes more sense in this context: recognizing the increasing risks of the weaponization of its trade dependence on China, it has naturally been reluctant to deepen that dependence further via a new trade agreement. And as other countries also try to decouple and diversify their economies from hub powers, India no longer looks like a complete outlier.

India’s narrative did not fundamentally change. It incorporated a further reason for exercising great restraint in market opening, without abandoning its earlier arguments on protecting its own poor. But in a world that is adapting to weaponized interdependence, India’s narrative no longer looks quite as anachronistic. Recall, for instance, India’s demands with reference to food security as part of the agriculture negotiations in the Doha negotiations. Today, as several countries besides India express similar concerns (e.g. in relation to food security, health security, security of communications and infrastructure sectors), India’s previous resistance to the old form of globalization begins to look almost pioneering.

## Conclusion

In this paper, I have highlighted the achievements and limitations in Modi’s economic policy. I have argued that while the Modi government has some important successes to its credit, particularly in certain aspects of its domestic economic policy, its agenda for the reform of foreign economic policy has been more cautious and is more closely aligned with India’s past. Even in the WTO—an institution that India has learnt to use rather effectively over the years—we do not see a transformative agenda espoused by Modi. Rather, we see some important continuities in the narrative that underpins India’s negotiation behaviour, that are also reflected in its bargaining strategy and coalition formation. This negotiation behaviour had, in previous years, won India the dubious distinction of being a difficult behaviour in the past; it had also generated mixed outcomes. Recent developments involving weaponized interdependence, however, now give a renewed energy and legitimacy to some of the concerns that India had voiced over the previous decades. Although India’s narrative has not changed in the Modi era—suggesting the limitations of Modi’s reformist agenda—the world has changed so much that some of India’s old narratives have acquired new relevance.

The world of weaponized interdependence is undoubtedly a much more precarious world, in comparison with the liberal order that had been inaugurated with the end of the Cold War. And unlike the conditions of dependency and asymmetric interdependence that developing countries have sadly been accustomed to endure, the deeply hierarchical networks that underpin weaponized interdependence make it even harder for countries that do not occupy network hubs to assert themselves. That said, as countries become more aware of the possibilities of weaponized interdependence, they become more adept at preempting it (e.g. Drezner 2019; Drezner et al 2021). Especially as countries attempt to decouple and diversify their supply chains, the negotiating space for developing countries like India increases (Narlikar 2021).

Whether India manages to have the last laugh in this new context will depend crucially on Modi’s next moves. There is a danger that in the attempt to restructure its value chains in the direction of self-reliance, India will end up repeating past follies. If Modi does indeed take the country down the route of self-sufficiency, he will undo the gains of the past nearly 30 years and impoverish his (still poor in per capita terms) country. Any kneejerk reactions to decouple from China will similarly be debilitating, given the dependence of the Indian economy on Chinese trade and investment. In a world of weaponized interdependence, economics and security come much closer together, and sometimes require trade-offs between prosperity and peace. To achieve the necessary balance between the two, foreign economic policy will probably need to become a more intrinsic part of the foreign/ security policy-making process, with attendant institutional implications and redistribution of tasks among ministries. Above all, to develop a constructive and sustainable version of Atmanirbhar Bharat, India will need to work closely with like-minded allies that share its values and can be relied upon, and not try to go it alone.

## Footnotes

1 It is worth recalling that prior to the work done in narrative economics, Goldstein and Keohane (1993) had developed a useful categorization of ideas in foreign policy: world-views, principled beliefs, and causal beliefs. There seems to be some overlap between Collier’s scheme and theirs. Identity would be a rough overlap with the ‘world-views’ box; norms would be a good fit with the ‘principled beliefs’ box; narratives would occupy a ‘causal beliefs plus’ box. But as argued in Narlikar (2020), narratives also go beyond ‘causal beliefs’. Goldstein and Keohane define causal beliefs as ‘beliefs about cause–effect relationships which derive authority from the shared consensus of *recognised elites*, whether they be village elders or scientists at elite institutions’. Narratives can be based on a shared political or epistemic consensus, but need not be. Some populist narratives in fact are formed explicitly *against* ‘recognised elites’. They can, in fact, originate in an urban myth or historical tradition, be true or false, be the work of individuals with no office or position, and ‘go viral’ through a variety of mechanisms used by individuals or groups of actors through word-of-mouth or social media—to thereby exercise an influence on foreign policy…. Narratives and frames are easier to operationalize and change than culture, identity, and norms.’

2 This concept shows some differences from President Trump’s ‘America First’. The Indian version is framed in terms of domestic governance, whereas the American version explicitly pits the interests of the American people against those of outsiders. For example, the BJP’s Election Manifesto (2014) had the following to say: ’India First simply means nurturing and protecting all the elements, which India is made of. It does not exclude anyone or anything—it only includes everything and everyone, which India is made of. It is complete India; without exclusion, without exception. It also means that whatever is in the interest of India will be in the interest of all the elements that India is made up of, including its citizens. The counter to the concept of 'India First' is what is happening today; appeasement of one, at the cost of the other, using Institutions for protecting the interest of a particular party or persons. It is reflected in the thinking that whatever is not in the interest of a party is not in the interest of India. Clearly, Governance on these lines is not in the interest of India.’ President Trump’s America First (2017) narrative was different. He asserted, at his inauguration, ‘We must protect our borders from the ravages of other countries making our products, stealing our companies, and destroying our jobs. Protection will lead to great prosperity and strength… America will start winning again, winning like never before. We will bring back our jobs. We will bring back our borders. We will bring back our wealth. And we will bring back our dreams.’

3 The furthest it has gone towards showing an independent position from China on SDT is via an additional proposal with some other developing countries (WTO 2019d). But this proposal does not does not represent any real dissociation of India from China on this issue.
